# Effects of the Aquatic Herbicide Diquat on Non-Target Aquatic Biota: A Mesocosm Study

**DOI:** 10.1007/s00244-025-01161-6

**Published:** 2025-09-26

**Authors:** R. L. Dalton, S. A. Robinson, A. J. Bartlett, V. Sesin, H. Ben Othman, D. J. Carpenter, A. Morrill, R. Prosser, J. Rohonczy, F. R. Pick

**Affiliations:** 1 Department of Biology, Carleton University, 1125 Colonel By Drive, Ottawa, ON K1S 5B6 Canada; 2 Department of Biology, University of Ottawa, 30 Marie Curie Private, Ottawa, ON K1N 6N5 Canada; 3 Wildlife and Landscape Science Directorate, Environment and Climate Change Canada, 1125 Colonel By Drive, Ottawa, ON K1A 0H3 Canada; 4 Water Science and Technology Directorate, Environment and Climate Change Canada, 867 Lakeshore Road, Burlington, ON L7S 1A1 Canada; 5 Institute of Environmental Sciences, University of Kaiserslautern-Landau, 76829 Landau, Germany; 6 School of Environmental Sciences, University of Guelph, 50 Stone Road East, Guelph, ON N1G 2W1 Canada; 7 Science Reporting and Assessment Directorate, Environment and Climate Change Canada, Gatineau, QC Canada

## Abstract

Invasive aquatic plants threaten the health of aquatic ecosystems, and demand for chemical control is likely to increase as nuisance levels are reached. Diquat is a contact herbicide registered in many countries to control invasive aquatic plants. The objective of our study was to assess the effects of the aquatic herbicide diquat (Reward®) on North American native and non-native plants, algal communities, an amphipod and an amphibian using outdoor mesocosms to simulate natural systems. Our experimental design included a control and five nominal concentrations of diquat ranging from 100% (18.3 L/ha; 1153 µg/L) to 6.4% (1.2 L/ha; 74 µg/L) of the label rate of a single diquat application. Effects of diquat were found to vary among study organisms. All four plant species were negatively affected at all concentrations, exhibiting either mortality or severe reductions in dry biomass (< 1% the biomass of the controls). In contrast, phytoplankton biomass increased 7 d following diquat application concomitant with significant changes in algal community structure. A concentration–response relationship was observed for amphipod survival (LC_50_ at 6 weeks = 155 µg/L) with 100% mortality in the highest treatment after two weeks. In contrast, diquat had a significant positive effect on tadpole survival, growth and development, possibly because of the higher algal biomass and decaying plant tissues. A lower label rate than currently recommended, at least in waterbodies with low turbidity, could provide effective control of target species while reducing effects on non-target biota.

**Supplementary Information:**

The online version contains supplementary material available at 10.1007/s00244-025-01161-6.

Native aquatic plant communities are essential components of healthy aquatic ecosystems, providing key habitat, refuge and food for aquatic species including fish (Thomaz and Cunha 2010). Invasive aquatic plant species, however, pose a significant threat to the health of these ecosystems and can severely impair waterways (Chambers et al. 1999; Boylen et al. 1999; Hummel and Kiviat 2004). On a global scale, invasive aquatic plants are expected to become increasingly problematic due to dispersion of plant species through shipping, recreational and irrigation activities (Hodkinson and Thompson 1997; Johnson et al. 2001; Thomaz and Cunha 2010). Fish may be particularly affected, and after habitat loss, the introduction of invasive species represents the greatest threat to fish species in North America (Lassuy 1995; Dextrase and Mandrak 2006). The effects of invasive plants are further compounded by nutrient enrichment, which can result in nuisance levels of native and invasive aquatic plants and algae.

As aquatic ecosystems increasingly face the combined stressors of invasive aquatic plants, eutrophication, and climate warming, there is likely to be a greater demand for chemical control options to manage aquatic invasive plants. Therefore, we may expect an increase in the use of currently registered aquatic herbicides, especially until new compounds are approved for usage. Many regulatory agencies around the world routinely re-evaluate registered products and reassess the potential risk of currently used aquatic herbicides, using new information and more sensitive and ecologically relevant methods. This is necessary to ensure that as science evolves, the use of registered products continues to be acceptable according to current standards (e.g., Health Canada 2021; US Epa 2025). For aquatic herbicides, it is important for regulatory agencies to understand their potential toxicity to non-target biota to protect freshwater biota of importance to ecosystem functions, as well as fishery resources. Currently, data on the effects of chronic, ecologically relevant concentrations of current-use aquatic herbicides on non-target biota are limited.

A relatively common aquatic herbicide used worldwide to control submerged, floating and emergent vegetation is diquat (Hussner et al. 2017); albeit, in 2019 its use was banned in Europe (Implementing regulation—2018/1532—EN—EUR-Lex). Commercial formulations of diquat are sold as diquat dibromide, which is highly soluble in water and readily dissociates to the diquat ion (US Epa 1995; Emmett 2002). At the time of this study, Reward® Aquatic Herbicide, containing the active ingredient diquat ((6,7-dihydrodipyrido[1,2-a:2′1’-c]pyrazinediium dibromide), was the only aquatic herbicide registered in Canada for controlling submerged aquatic plants in ponds, lakes, streams and canals (Breckels and Kilgour 2018). Diquat is a non-selective contact bipyridylium herbicide that functions by destroying cell membranes and disrupting photosynthesis by altering the flow of electrons in photosystem I (Dodge and Harris 1970; Emmett 2002). Diquat can be applied directly to water bodies by spraying, pouring it over the water surface or injecting it below the surface (Syngenta Canada Inc 2015). Diquat is effective at controlling submerged aquatic plants and some floating and emergent species (Emmett 2002; Syngenta Canada Inc. 2015; Hussner et al. 2017). Diquat breaks down quickly in the water column (half-life ranges from < 1 d to 4 d; Emmett 2002), and thus, repeat treatments (minimum 14-d intervals between treatments) may be necessary throughout a season to provide effective control of aquatic plants (Syngenta Canada Inc. 2015). Because diquat is inactivated quickly by binding to clay and organic matter (Simsiman and Chesters 1976), it has been approved for use in aquatic habitats as it is thought to pose minimal long-term risk to non-target aquatic biota. However, a variety of non-target biota may be exposed prior to adsorption to organic matter, and such exposure may jeopardize fish habitat and their prey. For example, the non-selectivity of diquat means that non-target native plants and algal species would also be affected, which could have direct consequences for the natural plant and algal community structure and thus overall ecosystem health (Peterson et al. 1997). Furthermore, freshwater species, including invertebrates and amphibians, are also at risk of exposure, with diquat being linked to potential toxic effects due to oxidative stress (Bouétard et al. 2012). Amphipods, for instance, are particularly sensitive to diquat in acute exposures; however, chronic effects have not been well studied (Emmett 2002). Only a few toxicity studies have been conducted on amphibians (e.g., frog tadpoles; Anderson and Prahlad 1976; Cooke 1977; Bimber and Mitchell 1978; Dial and Dial 1987), but based on these few studies, along with extrapolating results from chronic toxicity tests on fish, chronic exposure effects on amphibian behavior and other measures of stress may be expected (Emmett 2002). The effect of chronic exposure due to the slow release of diquat through decaying vegetation (Davies and Seaman 1968; Bugbee et al. 2020) has also not been considered. Therefore, the effects of chronic exposure on non-target aquatic plants, algae, invertebrates and amphibians are not clearly understood and require further study (Emmett 2002).

The objective of the current study was to characterize the lethal and sublethal chronic effects of exposure to direct application of the aquatic herbicide diquat (commercial formulation Reward®) to North American non-native plants (*Myriophyllum spicatum* L., *Hydrocharis morsus-ranae* L.), native plants (*Ceratophyllum demersum* L., *Elodea canadensis* Michx.), a crustacean invertebrate (the amphipod, *Hyalella azteca* (Saussure, 1858)), and an amphibian (Northern leopard frog, *Rana* [*Lithobates*] *pipiens* (Schreber, 1782)) using outdoor mesocosms to simulate natural environmental conditions. A mesocosm experimental approach was chosen because it lowers variability and increases replication compared to field studies and is more realistic than smaller-scale single species tests (Fraser and Keddy 1997). The mesocosms were maintained for several weeks to measure chronic, lethal and sublethal endpoints and to assess the persistence of diquat in surface waters. We selected these test organisms based on their ecological relevance to North American aquatic ecosystems. Specifically, native plants provide important habitat for breeding, foraging and escaping predators, and the amphipods and tadpoles/frogs provide an important food source for a variety of aquatic species (e.g., dragonfly larvae, fishes, birds and reptiles). We included two species that are non-native to North America as being representative of invasive species in aquatic ecosystems that are often targeted for removal.

## Materials and Methods

### Experimental Design

The mesocosm experiment was conducted during spring and summer at Carleton University’s outdoor research garden (Ottawa, ON, Canada) as described in Sesin et al. 2018. Briefly, the experiment consisted of a control and five diquat treatments, with five replicates each for a total of 30 experimental mesocosms. The diquat commercial formulation Reward® (240 g/L diquat dibromide; Syngenta Canada Inc; CAS: 85-00-7 (Syngenta Canada Inc. 2015)) was used. The five nominal test concentrations followed a geometric progression from 100% (18.3 L/ha; 1153 µg/L) to 6.4% (1.2 L/ha; 74 µg/L) of the label rate for waterbodies ≤ 1.5 m. A single application of diquat was applied after the mesocosms were established (July 20, 2016) and the mesocosms were monitored over a 6-week experimental period (July 20 to August 31, 2016). Mesocosm set up and take down were spread over 24 h (due to logistics) and are represented as day—1/0 and 41/42, respectively. Biota included a) North American native (*Elodea canadensis* and *Ceratophyllum demersum*) and non-native macrophytes (*Hydrocharis morsus-ranae* and *Myriophyllum spicatum*), b) natural communities of phytoplankton and periphyton, c) caged amphipods (*Hyalella azteca*) and d) northern leopard frog tadpoles (*Rana* [*Lithobates*] *pipiens*). Mesocosms were used to represent natural systems, and we used plants and sediment collected from nearby waterbodies as described in Sesin et al. (2018). Despite efforts to cure the sediment and careful inspection and cleaning of plant material, several unintended species were observed in the mesocosms including *Daphnia* spp. from tadpole stock tanks, dragonfly larvae from sediment and fish hatched from eggs on plants (*n* = 13). The dragonfly larvae, which can prey on tadpoles, were removed when observed.

### Mesocosm Design and Water Quality Monitoring

The mesocosms were plastic 378-L Rubbermaid® tanks 78 cm wide by 120 cm long and ~ 55 cm deep. Over 70 tanks were each filled with 280 L of City of Ottawa tap water (conductivity ~ 135 µS/cm with Ca concentrations ~ 12 mg/L) in early June, and the water was allowed to age for several weeks. These tanks included the experimental mesocosms (*n* = 30), as well as additional tanks for sediment aging, extra water, tadpoles and aquatic plants. Shade cloth (30%) was fitted over each mesocosm to prevent wildlife from entering the mesocosms and frogs from escaping. Air pumps fitted with air stones were added to each experimental mesocosm to ensure adequate oxygen throughout the experiment with minimal disturbance to the sediment. Data loggers (HOBO by Onset UA-002–64 pendant) were added to two randomly selected mesocosms per treatment for a total of 12 mesocosms to measure temperature and light continuously. As described in Sesin et al. (2018), temperature, pH and dissolved oxygen were measured with a YSI Professional Plus (Pro Plus; YSI Incorporated, Yellow Springs, OH, USA) sonde three times per week. Major fractions of nitrogen (nitrate/nitrite, ammonium/ammonia total Kjeldahl nitrogen) and phosphorus (soluble reactive phosphorus and total phosphorus) were measured in all 30 mesocosms on day 0 before diquat addition and at the end of the experiment (i.e., day 41). Nutrients were also measured in a subset of 12 mesocosms on day 7 and day 21. All nutrient analyses were conducted by the Robert O. Pickard Centre (Ottawa, ON, Canada), a Canadian Association for Laboratory Accreditation Inc. (CALA) accredited laboratory. Reporting detection limits were 0.003 mg/L for ammonium/ammonia, 0.02 mg/L for nitrate/nitrite and total Kjeldahl nitrogen, 0.002 mg/L for soluble reactive phosphorus and 0.005 mg/L for total phosphorus. Diquat was measured in all 30 mesocosms 1 h and 7 d after diquat application. Diquat was also monitored in a subset of 12 mesocosms (two per treatment) on days 1, 2, 4, 21 and 41 post-application. Collected water samples (1 L) were analysed for diquat by Caduceon Environmental Laboratories (Kingston, Ontario), a CALA accredited laboratory with a reporting detection limit of 5 µg/L, as described in Sesin et al. (2018).

### Plants and Sediment

Full details of plant and sediment setup and conditions are described by Sesin et al. (2018). Briefly, OECD sediment (OECD 2014) was prepared and mixed with the natural sediment using a portable cement mixer (ratio of 80% OECD to 20% natural sediment by volume). Six plastic pots (25.4 cm diameter) with drainage holes were prepared for each experimental mesocosm. Pots were lined with landscape cloth and were filled following a layering pattern of stone (900 mL) and sand (500 mL) and then sediment (2 L), with a final covering layer of sand (500 mL) to keep the sediment in place. The pots were acclimatized and aged by submerging them in tanks that had previously been filled with City of Ottawa tap water for at least 10 d to minimize plumes of nutrients or unwanted soil organisms in experimental mesocosms.

Following sediment aging, all plants were added to the mesocosms approximately 21 d prior to the addition of diquat to allow them to become established. Loading densities for the rooting species, *E. canadensis* and *M. spicatum,* consisted of 30 apical stems (12 cm long), planted 10 per pot for three separate pots per species for each mesocosm. Additionally, 30 apical stems of the submerged, non-rooting species *C. demersum* (12 cm long) and 10 individual *H. morsus-ranae* plants (with 5–11 developed leaves) were added to each mesocosm. Further details are available in Sesin et al. (2018).

After the addition of diquat, plants were monitored weekly for signs of negative effects of diquat (i.e., chlorosis and necrosis of tissues). At the end of the experiment, plant survival was recorded, and dry shoot mass was assessed as an endpoint for each species.

### Algae

Phytoplankton communities colonized the mesocosms naturally, arising from algae on the plants and the natural sediment. We estimated algal pigments within the mesocosms with a submersible FluoroProbe (BBE Moldaenke), through a collaboration with the Université du Québec à Montréal (B. Beisner), on days—1, 2, 7, 21 and 41. The FluoroProbe measured in vivo chlorophyll a (a proxy for total algal biomass), along with the relative contribution of major groups (e.g., chlorophytes, cyanobacteria, diatoms, cryptophytes) based on pigment fluorescence signals. For each mesocosm and timepoint, means were calculated from triplicate measurements, each representing approximately 10 readings. Water samples were also collected for phytoplankton identification and enumeration on days—1, 2, 7, 21 and 41. A 100-mL sample of water was collected from mid-water column for each treatment mesocosm. Immediately after collection, acid Lugol’s solution was added to the sample to preserve the phytoplankton cells at a final concentration of 1% glacial acetic acid. A subsample of 10 mL was allowed to settle for 24–48 h in a sedimentation chamber following the Utermöhl method (Utermöhl 1958) and was then observed under a Zeiss Axio A1 microscope at 200X and 400X magnification. One to two transects of the sedimentation chamber were examined, and at least 300 cells in each sample were identified and counted. Phytoplanktons were identified to the highest taxonomic resolution (~ genus/species) possible, and cell densities, along with biomass (mg/m^3^), were calculated using ALGAMICA version 4.1 software (Gosselain and Hamilton 2000).

Changes in community structure in relation to treatment were assessed through analyses of both the Shannon diversity index and algal taxa composition. Relationships between Shannon diversity index and experimental treatment concentrations (coded as a factor) were analyzed using linear models that included an interaction between the experimental treatment and the treatment day (either day 0 (i.e., pre-dosing), 7 or 41). We used non-metric multidimensional scaling (NMDS) and permutational multivariate analysis of variance (PERMANOVA) to test for effects of the different diquat treatments on algal taxa composition on each of the three experimental days, based on Bray–Curtis dissimilarities between tanks, calculated from algal cell counts. In the NMDS analysis, a stress value less than 0.1 was considered indicative of a good (non-arbitrary) ordination of the data. PERMANOVA tests were preceded by tests of the homogeneity of group dispersions (betadisper() function in the R package vegan; Oksanen et al. 2022) to ensure that any significant PERMANOVA result could be interpreted as arising from location effects (i.e., differences in means among the groups) rather than dispersion effects (differences in variation; Anderson 2001). All statistical analyses were conducted in the statistical programming language R (R Core Team 2022), and algal composition analyses relied on the R package vegan (distance calculations, NMDS, tests of the homogeneity of groups dispersions, PERMANOVA; Oksanen et al. 2022). Post hoc contrasts of Shannon diversity linear model predictions were computed using the marginaleffects R package (Arel-Bundock 2023).

### Amphipods

The amphipod *Hyalella azteca* was cultured at Environment and Climate Change Canada’s Canada Centre for Inland Waters (Burlington, ON) and transported to Carleton University at the end of June 2016. Amphipods were cultured in 2-L polypropylene containers according to the methods described in (Borgmann and Munawar 1989). Cultures were maintained at 25 °C with a photoperiod of 16 h light:8 h dark, and amphipods were fed ground TetraMin (Tetra GMBH, Melle, Germany) fish food flakes shifted through a 500-µm screen. Juvenile amphipods were removed from the breeding containers weekly for use in exposures.

Amphipod cages were constructed of clear acrylic tubing (total length 8 cm, outside diameter 7.6 cm) and sealed at each end with 300-µm Nitex mesh (Bartlett et al. 2016). Three cages were deployed per mesocosm, each cage containing 20 amphipods (1–2 weeks old), a square of 5 cm × 5 cm cotton gauze, and 10 mg ground TetraMin. Cages were gently submerged in the mesocosms, and air bubbles removed by tapping the cages or using a disposable plastic pipette. Cages were suspended approximately 15 cm from the surface of the water by tying one end of a Dacron fishing line to the cage and the other end to a fishing bobber. Amphipods were acclimated in the mesocosms for approximately three weeks prior to dosing.

Cages were examined weekly to ensure that the mesh was not fouled and that water was flowing freely through the cage; the mesh was cleaned by scrubbing gently with a toothbrush if necessary. Cages were removed from the mesocosms after two weeks of acclimation to assess survival, to ensure the cages were clean and undamaged and to feed the amphipods. The number of surviving amphipods (F0) was recorded and returned to the cleaned cage with fresh TetraMin (15 mg/cage) and a fresh piece of cotton gauze. As some mortality occurred during the 3-week acclimation period, amphipod cages were removed one day prior to dosing, and the three original replicates per mesocosm were condensed into two replicates per mesocosm, each containing 20 amphipods, a fresh piece of cotton gauze, and 15 mg TetraMin.

Following diquat dosing of the mesocosm, cages were examined weekly as described above and were removed every two weeks to assess survival and reproduction, to ensure the cages were clean and undamaged, and to feed the amphipods. Numbers of surviving adult amphipods (F0) and mating pairs were recorded and returned to the cleaned cage with fresh TetraMin (30 mg/cage) and a fresh piece of cotton gauze. The number of juvenile amphipods (F1) was recorded, but the juveniles were then discarded to reduce the density of amphipods in the cage during the exposure period. At the end of the 6-week exposure, amphipods were removed from each cage. Numbers of adult amphipods (F0) and mating pairs were recorded, and then the adults were rinsed three times in clean culture water and weighed as a group to obtain total wet weight per cage. The number of juvenile amphipods (F1) was recorded, and then the juveniles were discarded.

We modeled the survival of amphipods as a binomial response dependent on (log_2_-transformed) diquat concentrations following a quadratic polynomial relationship on the logit scale, as we suspected survival increased at lower concentrations and decreased at higher concentrations. We chose a quadratic polynomial rather than applying alternative non-linear formulations of survival (e.g., direct mathematical models of hormesis; Zhang and Lin 2020) for several reasons: We wanted to model individual-level amphipod survival probability directly using the appropriate (binomial) response distribution, through a linear model on the logit scale; we needed to directly compare the model to simpler logistic regressions without the quadratic term; and also because models required the incorporation of observation-level random effects to account for extra variation related to the individual tanks. This modeling approach additionally allowed us to calculate concentrations of interest directly from the model as parameters, with their uncertainties, using solutions to the quadratic equation (e.g., the lethal concentrations, the diquat concentration at which the survival probability returns to the control value after the initial increase). Amphipod survival models were coded in the Bayesian statistical programming language Stan (Stan Development Team 2024), in part to allow the direct quantification of concentration-related parameters (e.g., median lethal concentration (LC_50_)) with their uncertainties, and were run through the cmdstanr R interface (version 0.5.3; Gabry and Češnovar 2022). Details of Bayesian amphipod survival model formulations and evaluations of model fit are provided in the Online Resource (Online Resource 1). Diquat concentrations were increased by one prior to log_2_-transformation so that expected survival probability at the model intercept corresponded to control concentrations. Models were compared using exact leave-one-out cross-validation, based on log pointwise predictive densities (Vehtari et al. 2017). Errors reported on estimates from the Bayesian models are 95% credible intervals, calculated as highest density continuous intervals.

Growth (mg wet weight/amphipod at 6 weeks) and reproduction data (conditional on survival; number of juveniles/amphipod at 6 weeks) did not follow concentration–response relationships, and no amphipods survived in the 1153 µg/L treatment at 6 weeks. Therefore, effect concentrations of 50, 25, and 10% (EC50s, EC25s, and EC10s) could not be determined. Log_2_-transformed biomass values were compared using ANOVA because linear mixed-effects models with tank-level random effects would not converge. Post hoc comparisons of transformed growth among treatment groups were conducted using Tukey’s HSD test. To test for differences in the proportions of juveniles among the treatments, we fit binomial generalized linear mixed models (GLMMs) with observation-level random effects to account for overdispersion. The GLMM with an effect of treatment concentration (increased by one and log_2_-transformed) was compared, using AICc values (Akaike’s Information Criterion corrected for small sample sizes), to a null model without an effect of diquat. GLMMs were fit using the lme4 package in R (Bates et al. 2015).

### Amphibians

Adult northern leopard frogs (*Rana* [*Lithobates*] *pipiens*) were collected from the wild and bred at the University of Ottawa (following (Trudeau et al. 2013). The resulting five egg masses were reared in mesocosms to free-swimming tadpoles (i.e., Gosner stage 25; Gosner 1960). Fifteen tadpoles between Gosner 25 and 27 were added to each mesocosm on July 12, 2016, and were left to develop for 53 d. Throughout the experiment, as tadpoles completed metamorphosis (i.e., Gosner 45 or 46), they were removed from the tanks and processed. At the end of the experiment (or earlier as individuals reached metamorphosis), tadpoles/metamorphs were processed following similar protocols as described in Robinson et al. (2017), which included collecting a wet weight, taking a photograph for later body length measurements using image analysis software, euthanasia, and dissection to weigh and collect liver samples for oxidative stress measures. We measured the concentration of 4-hydroxylnonenal (HNE) protein adducts and protein carbonyl to assess the amount of lipid peroxidation or protein carboxylation, respectively, after exposure to diquat following methods described in Robinson et al. (2021). Briefly, liver tissue from all tadpoles in a mesocosm (*n* = 11–12 tadpole livers) were pooled and homogenized, mixed with solvents and distributed to 96-well enzyme-linked immunosorbent assay (ELISA) plates for visualization and quantification via spectroscopy. Tadpole livers were pooled to provide sufficient liver tissue (~ 0.1 g of tissue) for the analyses.

We modeled tadpole survival until the end of the experiment using GLMMs with a binomial response, including experimental tank as a random effect. The relationship between (log_2_-transformed) initial diquat concentration (+ 1 μg/L) and tadpole survival on the logit scale was modeled as following either a linear or quadratic polynomial trend, somewhat similarly to the amphipod survival analysis, and we compared these models and a null model (no treatment effect) using AICc values. Models with ΔAICc < 2 were considered as providing essentially equivalent fits to the data (Burnham and Anderson 2002). The quadratic polynomial relationship between transformed diquat concentration and logit-transformed survival probability was included to test whether the effect of diquat on amphibian survival changed with increasing diquat concentration (e.g., survival probability increasing at lower concentrations but decreasing at higher concentrations; a hormetic effect, possibly indirect). We could not test the potential additional effects of tadpole sex, Gosner stage, or liver mass on survival as models would not converge with these added predictors regardless of whether the variables were standardized; models would also not converge whenever the random effect of experimental tank was nested within block. This was likely in part due to the overall high frequency of tadpole survival (85.8%), meaning there was little variation in survival to explain after accounting for tank and treatment effects (i.e., estimating 30 random intercepts and one slope parameter). GLMMs were fit using the lme4 package in R, with the BOBYQA optimization algorithm (Powell 2009; Bates et al. 2015).

In addition to tadpole survival, we analyzed various responses in surviving tadpoles relevant to life history traits and fitness, including developmental rate (average Gosner stages incremented per day), mass, sex ratios and hepatosomatic index (HSI), using linear mixed-effects models (LMMs, for all of these responses except sex ratios and oxidative stress responses) and GLMMs (sex ratios; binomial response of female vs. not, i.e., vs. male or unknown). The LMM and GLMM analyses all included experimental tank as a random effect (models with tank nested within block would not converge). Developmental rate, mass and HSI responses were all log_2_-transformed prior to modeling to ensure normality of model residuals, and therefore, estimated effects should be interpreted as relating to doublings of these responses. In addition to log_2_-transformed initial diquat concentration, models of HSI and sex ratios included Gosner stage as a potential predictor. Tadpole sex was not included as a predictor with Gosner stage in models of mass and HSI since the recorded sex of the tadpole carries significant information about development (tadpoles earlier in development were far more likely to be categorized as “sex unknown”). Analysis of tadpole sex ratios (female vs. not) were restricted to tadpoles at Gosner stages ≥ 36, as morphological sex is difficult to assess with confidence at earlier stages (Hogan et al. 2008). A potential polynomial relationship with Gosner stage was also considered in models of tadpole mass and HSI due to expected non-linearities in these relationships. GLMMs were fit using the lme4 package in R and compared based on AICc values (Bates et al. 2015). Because oxidative stress response measures (i.e., HNE and protein carbonyl) were based on tadpole samples pooled within mesocosms and because data did not meet parametric model assumptions, these two responses were compared between treatments using non-parametric Kruskal–Wallis tests. HNE and protein carbonyl mean values are reported with bias-corrected and accelerated 95% confidence intervals as their distributions were not normal, even after log-transformation.

## Results

### Mesocosm Abiotic Conditions

Continuous temperature measurements were similar among treatments, ranging over time between 18 and 36 °C (Fig. S1) with an overall decline mid-way through the 6-week experiment (July 20–Aug 31). Light levels reaching the bottom of the tanks did not differ significantly among treatments over time, although one tank at the second highest diquat concentration had somewhat lower light levels compared to the other four replicates (Fig. S2). The gradual decline in daily intensity is consistent with shortening of daylight hours through late summer at this latitude (i.e., 45.3872° N).

**Fig. 1:**
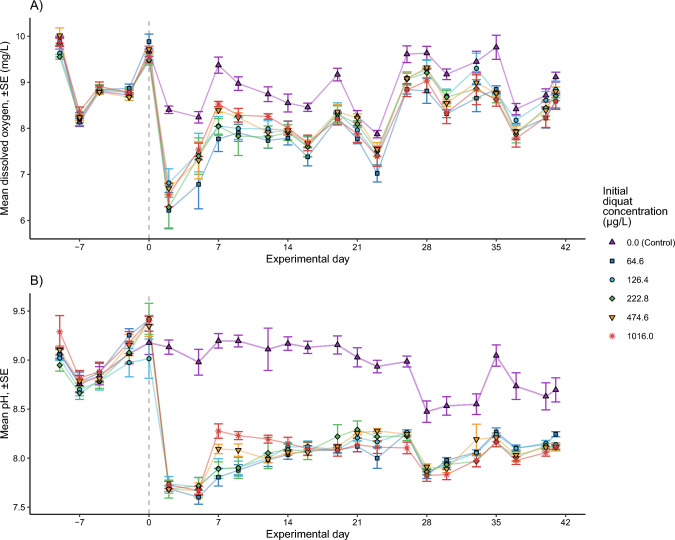
Mean (± standard error) dissolved oxygen (**A**) and pH (**B**) recorded in mesocosms across the course of the experiment for different diquat treatments. The vertical dashed line indicates the date that diquat was applied. After the application of diquat, both dissolved oxygen and pH decreased relative to the controls across all treatments, though differences among the diquat treatments (i.e., separate from the control) were generally non-significant (*p*-values < 0.001 for all treatment-vs.-control comparisons from linear mixed-effect models with post-diquat administration date as random effect, after controlling for multiple comparisons using the Holm–Bonferroni method). The legend presents diquat treatments as mean concentrations, measured one hour post-diquat application (i.e., initial diquat concentration)

In contrast, dissolved oxygen (DO) varied more across treatments, with the highest concentrations seen in the control over time. DO decreased sharply relative to controls in all treatments following application, from ~ 9.5 to 6.2–6.8 mg/L DO (Fig. 1A). DO partially recovered in the diquat treatments over time and was similar between the control and treatments by the end of the experiment. Likewise, pH showed a sharp decline relative to the control immediately after application, from ~ 9.2 to ~ 7.6, but never fully recovered to control levels by the end of the 6-week experiment (Fig. 1B).

Nutrients analyses of the mesocosms showed similar concentrations across the control and treatments prior to diquat applications, then a sudden increase in both total phosphorus and total Kjeldhal nitrogen (essentially equivalent to total nitrogen as nitrate concentrations were close to detection) in the treatments, quasi in proportion to the diquat applications (Fig. S3). Nutrient concentrations of the diquat-treated mesocosms then declined gradually, reaching similar concentrations to controls by the end of the experiment, particularly for total phosphorus.

### Diquat Concentrations

**Fig. 2:**
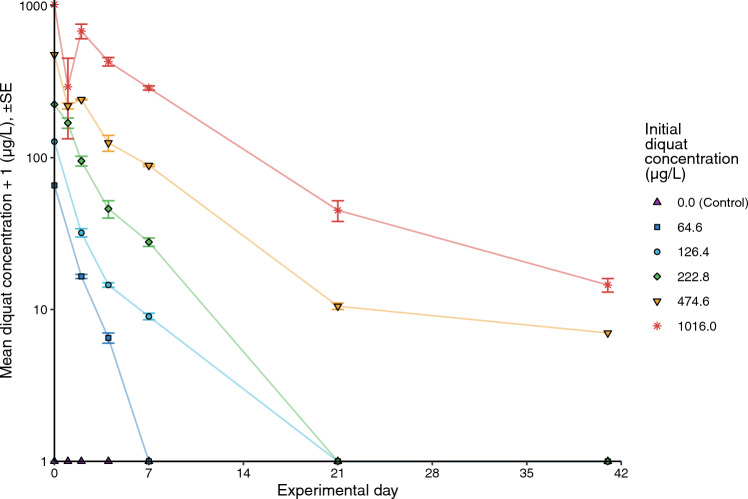
Average measured concentrations of diquat (+ 1 µg/L) in mesocosms treated with a single application of diquat on July 20, 2016 (day 0). The legend presents diquat treatments as mean concentrations, measured one hour post-diquat application (i.e., Initial diquat concentration). Note that the Y-axis is on the log_10_ scale. All points on the X-axis represent values below the reporting limit (< 5 µg/L). The applied concentration in the highest concentration treatment group is the application rate. Points without standard errors (SEs) represent cases where the averaged measured values were identical. Mean concentrations were calculated from five or fewer observations. Error bars represent standard error around each mean

Diquat concentrations measured in the mesocosms one hour after dosing were 84.0 ± 2.0% (mean ± standard error; *n* = 25) of nominal concentrations, with a range of 70.5 to 108.4% (Fig. 2). Diquat declined rapidly, with ~ 50% loss within two days for the three lowest nominal concentrations (74, 147 and 291 µg/L) and within two to four days for the two highest nominal concentrations (579 and 1153 µg/L). Diquat concentrations were below detection limits (5 µg/L) throughout the experiment for the controls. Diquat in the three lowest test concentrations was also below detection at 21 d post-dosing, but it persisted at trace and low concentrations in the mesocosms treated with 579 and 1153 µg/L diquat throughout the 42-d experimental period (Fig. 2).

### Plants and Algae

Both North American native and non-native plants were severely affected in all treatments, including the lowest application rates of 6.4% of the label rate (equivalent to 74 µg/L nominal diquat here or 64.6 µg/L measured diquat), with chlorosis followed by necrosis beginning in the first few days post-diquat application, and little to no recovery observed over the 42-d test period. Full macrophyte comparisons are available in Sesin et al. (2018). Notably, both native and non-native plants in the control mesocosms appeared healthy (e.g., green with new growth) throughout the experiment (mean (± SD) dry biomass per tank: *C. demersum* (native) = 6.27 ± 1.33 g; *E. canadensis* (native) = 5.53 ± 1.03 g; *H. morus-ranae* (non-native) = 1.31 ± 0.32 g; *M. spicatum* (non-native) = 4.42 ± 2.34 g). Following diquat exposure, both non-rooted plants, *C. demersum* (native) and *H. morsus-ranae* (non-native) experienced complete mortality in all diquat treatments by the end of the experiment. Furthermore, only two small living remnants of *M. spicatum* (non-native, rooted plant) were observed in one 64.6 μg/L diquat treatment (dry biomass of surviving plants = 0.0305 and 0.0005 g). In contrast, there were some surviving *E. canadensis* (native, rooted plant) across all diquat concentrations, except in the 222.8 μg/L treatment where all individuals died; however, the dry biomass of these surviving plants was significantly reduced (< 0.5% the size of the corresponding controls). Additionally, a species that was not part of the study design was observed in some treated pots (*n* ~ 30) and was identified as a macroscopic alga (Characeae) that was common in the wetland where the natural sediment was collected.

**Fig. 3:**
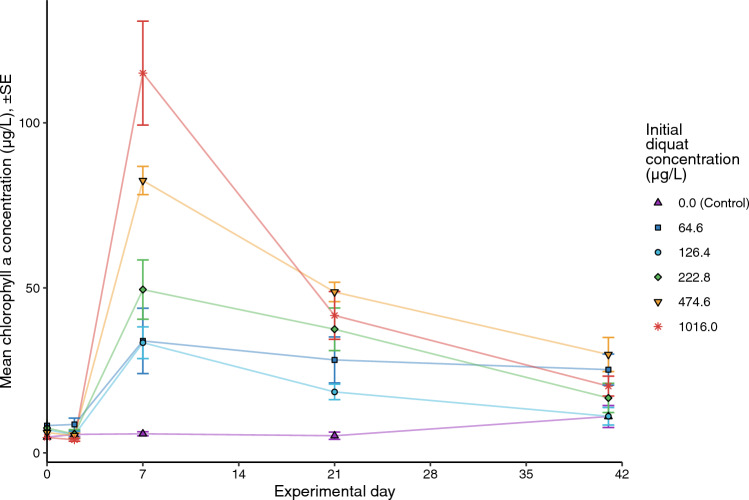
Average concentrations of phytoplankton chlorophyll a (µg/L) in mesocosms treated with a single application of diquat on July 20, 2016 (day 0), as estimated using in situ pigment fluorometry. Treatment-level means for each timepoint are calculated from replicate-level mean measurements. The legend presents diquat treatments as mean concentrations, measured one hour post-diquat application (i.e., initial diquat concentration). Error bars represent standard error around each mean

Phytoplankton chlorophyll *a* was higher in the treated mesocosms after the addition of diquat with the treated mesocosms differing from the controls in a clear concentration-dependent manner by day 7 (Fig. 3). Following day 7, chlorophyll concentrations declined in all treated mesocosms, and by the end of the experiment were more similar across treatments while remaining slightly more elevated than in control mesocosms. Overall, there was a significant positive correlation between chlorophyll a and phytoplankton carbon biomass (linear model *R*^2^ = 0.55, *p*-value ≪ 0.001) indicating good correspondence between FluoroProbe and microscopy estimates of phytoplankton biomass. Across all treatments, phytoplankton communities were dominated by green algae over the course of the experiment (91 ± 7.4% total chlorophyll a) based on the FluoroProbe results. Cyanobacteria, diatoms and cryptophytes represented 1.6 ± 2.1%, 6.6 ± 4.8% and 1.0 ± 1.2% of the communities, respectively. The phytoplankton community analyzed via inverted microscopy corroborated these results: biomass was dominated by chlorophytes across all treatments.

**Fig. 4:**
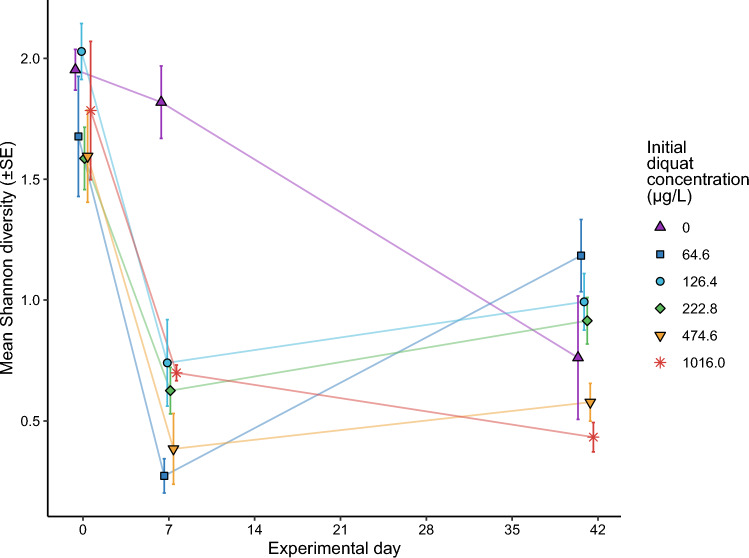
Mean observed phytoplankton Shannon diversity index for each initial diquat concentration at days zero, seven, and 41 of the experiment. Points represent individual experimental mesocosms and are slightly shifted horizontally in different directions to help reduce overlap, not because observations were made at any times other than the three indicated days. Lines connecting points are to aid visual comparisons of mean Shannon diversities between times for individual treatments; phytoplankton mean diversity between these observations are unknown. The legend presents diquat treatments as mean concentrations, measured one hour post-diquat application (i.e., initial diquat concentration). Error bars represent standard error around each mean

Based on microscopy analyses, the algal community’s Shannon diversity index changed significantly over time and in response to different diquat treatment concentrations (Fig. 4). Comparisons of linear models indicated an interaction between diquat treatment concentration and observation day (Table S1): algal diversity was similar in all tanks prior to the addition of diquat (day 0) and then plummeted across all diquat treatments compared to the control (day 7; Fig. 4). For example, the best-fitting linear model predicted that average algal Shannon diversity index was significantly lower in all the treatment groups relative to the control at day 7 (all comparison *p*-values < 0.001). However, by day 41, the diversity observed in the control treatment had decreased to levels similar to most of the other treatments.

**Fig. 5:**
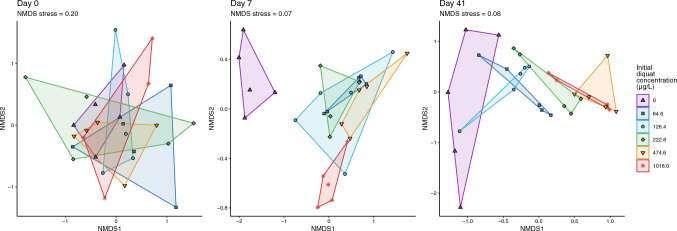
Phytoplankton assemblage non-metric multidimensional scaling (NMDS) projections based on Bray–Curtis dissimilarity matrices at three time points relative to experimental treatment with diquat. Low stress values (< 0.1) on days seven and 41 indicate good (non-arbitrary) ordinations. Points represent individual experimental tanks, and polygons are the minimum convex hulls for tanks in each treatment. The legend presents diquat treatments as mean concentrations, measured one hour post-diquat application (i.e., initial diquat concentration)

At the start of the experiment (day 0), there were no detectable differences in algal community composition among treatment groups, as indicated by Bray–Curtis dissimilarities (PERMANOVA *p* = 0.961). However, by day 7 and day 41, significant differences had emerged (day 7: *p* = 0.001, *R*^2^ = 0.51; day 41: *p* = 0.001, *R*^2^ = 0.37). These results reflect meaningful shifts in community structure, as tests for homogeneity of group dispersions were non-significant, confirming that the observed differences were not due to variation in within-group spread. While the non-metric multidimensional scaling (NMDS) projection on day 0 had a stress value of 0.20 which is too high to be considered reliable, by day 7 the projections showed clear separation between the control and diquat-treated groups, with low stress (0.07; Fig. 5). However, the treatment groups exposed to non-zero diquat concentrations did not show strong separation from one another at this timepoint. Within these treatments, *Chlorella* spp. and *Scenedesmus quadricauda* emerged as dominant taxa, comprising over 40% of total phytoplankton biomass in the three lower diquat treatments and over 90% in the two highest treatments. By day 41, a positive trend was observed between diquat concentration and the distance of each treatment group from the control in NMDS space (Fig. 5). This suggests that, even though the Shannon diversity index did not differ significantly at this timepoint, the overall community composition continued to diverge from the control, with greater differences seen at higher diquat concentrations.

### Amphipods

**Fig. 6:**
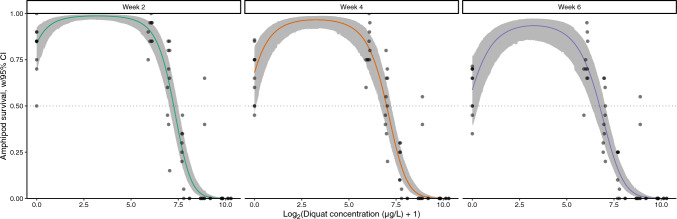
Changes in the proportion of amphipods (*Hyalella azteca*) surviving in each tank at weeks two, four, and six following experimental treatment with diquat. Colored lines and shaded regions represent best-fitting Bayesian model expected (median) survival and 95% credible intervals, respectively. Points represent observed proportions of amphipods surviving in each tank. Credible intervals are calculated as highest density continuous intervals. Diquat concentration values were increased by one μg/L prior to log_2_-transformation; a value of zero on the X-axis therefore represents the control treatment. Model predictions are conditional, i.e., they represent expectations for a single (average) tank

Control survival in amphipod cages exceeded the performance criteria recommended by (Borgmann 2002) for water-only laboratory tests of < 10% mortality per week or 65% survival in a 4-week test: Average control survival was 82, 67 and 60% following 2, 4 and 6 weeks of exposure, respectively (Fig. 6, Table S2). Complete mortality occurred after a 2-week exposure at the highest test concentration (Fig. 6).

Statistical models analyzing amphipod survival showed stable convergence throughout Markov chain Monte Carlo (MCMC) sampling, had well-mixed MCMC chains (all *R̂* values < 1.01), and produced parameter estimates with effective sample sizes ranging from 613 to 6,607. Model comparisons supported a non-linear (quadratic) relationship with diquat concentrations on the logit scale (Table S2). Specifically, survival initially increased at the lowest tested concentration of diquat (log_2_-transformed) but decreased at higher concentrations. This quadratic model outperformed both a simpler logistic regression and a model without diquat effects (see Table S2). Predicted survival from the best-fitting model closely matched observed survival across tanks (Fig. 6). Although the overall survival rate declined between week two and week six (e.g., control group survival was lower at week six by 0.245 on the logit scale; 95% CI − 0.482–− 0.023), the concentration of diquat needed to cause a specific reduction in survival did not change over time (LC_50_, LC_25_, and LC_10_ remained consistent; Table S3). The limiting concentration of stimulation, where survival returns to control levels, was estimated to fall between 46.85 and 136.07 μg/L of diquat and was consistent across durations (Table S3). The peak survival occurred at diquat concentrations between 7.97 and 12.89 μg/L and was similar across all exposure durations.

Beyond effects on survival, amphipod growth and reproduction were not negatively affected by exposure to diquat but instead increased relative to controls across all diquat concentrations at week six following the initial treatment (Fig. S4 – S5). The ANOVA comparing log_2_-transformed growth across treatments was significant (F_4,33_ = 19.27, *p* = 2.93 × 10^–8^), with post hoc comparisons indicating not only that increased growth occurred in all diquat treatments, but also that both the 126.4 and 222.8 μg/L treatments showed increased growth compared to the 64.6 μg/L treatment (Fig. S4). The binomial reproduction GLMM (proportion of juveniles, conditional on survival at 6 weeks) which included a treatment effect provided a better fit than the null model (ΔAICc = 6.32), with the predicted proportions of juveniles per cage increasing with increasing diquat (Fig. S5; Table S4).

### Amphibians

**Fig. 7:**
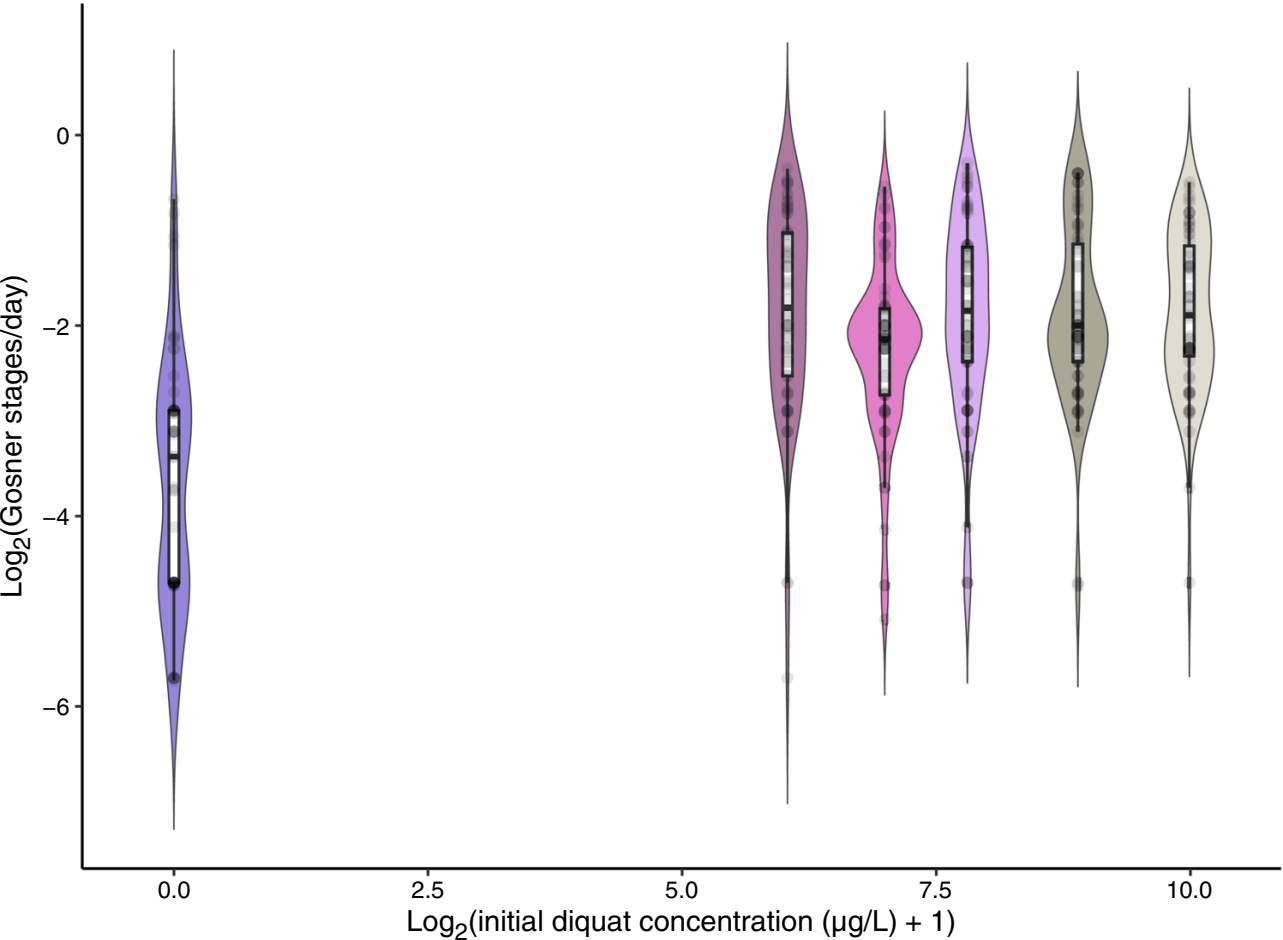
Log_2_-transformed development rate (increase in Gosner stages/day) of tadpoles (*Rana* [*Lithobates*] *pipiens*) surviving until the end of the experiment, following treatment with diquat. Points represent the observed transformed development rates of each tadpole. Points are partially transparent to help distinguish regions of overlap, and filled mirrored curves show point densities. Diquat concentration values were increased by one μg/L prior to log_2_-transformation; a value of zero on the X-axis therefore represents the control treatment

Several apparent positive effects of diquat exposure were observed among tadpoles. The best-fitting model showed that survival increased with higher diquat concentrations (Table S5). In an average tank, survival rose from 77.5% in the control group (95% CI 67.1%–87.9%) to 90.0% at the highest diquat concentration (95% CI 85.7–94.3%). Similarly, tadpole development rates increased with exposure. Tadpoles in the highest diquat treatment progressed through Gosner stages at a rate of 0.333 stages per day (95% CI 0.274–0.405), compared to just 0.092 stages per day in the control group (95% CI 0.067–0.125; Table S5; Fig. 7). However, this increase appeared to be consistent across all diquat treatments, suggesting that any exposure above zero accelerated development, rather than there being a continuous concentration-dependent effect. Tadpole mass also increased with diquat exposure even after accounting for the natural rise and fall in mass across developmental stages (Table S5; Table S6). Specifically, each doubling of diquat concentration was associated with a 2.29% increase in tadpole mass (95% CI 0.85–3.75%) in an average mesocosm. In contrast, no effects of diquat were detected on tadpole sex ratios or hepatosomatic index (HSI; Table S5). Models of sex ratio only converged when tank-level variation was removed, and even then, no relationship with diquat concentration was found (Table S5). Similarly, while HSI increased with Gosner stage, indicating that liver size relative to body size grew with development, there was no evidence that diquat exposure influenced this metric (Table S5). Finally, no significant differences were found in oxidative stress markers between control and diquat treatments. Due to sample pooling, each treatment group had only five observations. For both HNE (Kruskal–Wallis *p*-value = 0.32; mean HNA-BSA = 323.8 µg/g ww; 95% CI 237.6–456.3) and protein carbonyl (*p*-value = 0.19; mean protein carbonyl = 1.19 nmol/mg protein; 95% CI 1.03–1.35), results indicated no measurable impact of diquat exposure*.*

## Discussion

We found that all tested species were affected by diquat at or below label application rates, with: aquatic plants, algae and amphipod survival being negatively affected; growth and reproduction of amphipods increasing with diquat concentration; and tadpoles benefiting from the resulting increase in food resources. Furthermore, diquat persisted to the end of the experiment in the two highest treatments, which was longer than expected in this system, possibly from release of macrophyte bound diquat during plant degradation, static exposure conditions or low availability of sediment/suspended particles binding surfaces. This suggests that a prolonged exposure (i.e., 41 d) could occur in non-turbid aquatic ecosystems treated with diquat at 100% (1016 µg/L) and 50% (474.6 µg/L) of the label application rate for waterbodies ≤ 1.5 m.

### Plants and Algae

Diquat was highly effective at controlling both North American native and non-native plant species. Diquat is non-selective, and several test species (*C. demersum*, *Elodea spp*., and *Myriophyllum spp*.) are listed as potential target species on the Canadian label for Reward® (Syngenta Canada Inc. 2015). Of note is that non-native *H. morsus-ranae* was also effectively controlled by diquat. This species is one of the principal invasive wetland species in Canada with no known control measures other than physical removal of small infestations (White et al. 1993), and it forms dense floating mats that create anoxic conditions when the plants senesce, which often results in fish kills (personal communication, Naomi Langlois-Anderson, South Nation River Watershed Conservation Authority). Little recovery of plant biomass was observed over the 6-week experimental period, suggesting that a single application of diquat may provide effective control of target plant species throughout a growing season in a closed system. *E. canadensis* and *H. morsus-ranae* are effectively controlled by diquat at nominal concentrations ranging from 74 to 1153 µg/L under greenhouse conditions (Sesin et al. 2018), which is in agreement with the current study. Experiments at a lower range of nominal concentrations (4.7 to 74 µg/L) demonstrated that *M. spicatum*, *E. canadensis* and *C. demersum* are highly sensitive to diquat with calculated 50% effective concentrations for some species and endpoint combinations below the analytical detection limit of 5 µg/L (Sesin et al. 2018). Therefore, based on the present study and the work by Sesin et al. (2018), it may be appropriate to consider lowering application rates, given effective macrophyte control occurred at concentrations ~ 0.5% of the current label rate in an experimental closed system. However, further studies would be beneficial to determine efficacy across systems with varying concentrations of suspended sediment in the water column and varying percentages of clay in the sediment.

There was some evidence of growth of Characeae in the diquat-treated mesocosms following the die-off of the macrophytes. These plant-like macroscopic algae likely originated from the natural wetland sediment. This family is resistant to diquat (Syngenta Canada Inc. 2015), and therefore, in aquatic systems where Characeae thrive, treatment with diquat may lead to an overabundance of these resistant species at the expense of other macrophytes. Interestingly, the starry stonewort (*Nitellopsis obtusa*)*,* a Characeae species that is highly invasive in North America (Larkin et al. 2018), was controlled effectively under laboratory conditions by diquat levels within the range of those tested in the present study (190 and 370 µg/L; Wersal 2022), while the same concentrations were ineffective in the field (Carver et al. 2022). Hence, testing with only a few species may not be predictive or protective for ecosystems, and field testing would likely be required to verify the extent to which the label rate could be reduced in an open system/actual field conditions.

After the die-off of plants, the most visible effect of diquat on the mesocosms was the pulse in phytoplankton biomass which occurred within four days following the diquat applications (Fig. 3). High concentrations of chlorophyll a, greater than those typically associated with algal blooms (> 20 µg/L; Pick 2016), were found in all diquat treatments soon after application. The most plausible explanation is that the senescence and decomposition of the plants in the diquat treatments led to the measured increase in nutrients (Fig. S3) and subsequently stimulated rapid phytoplankton growth (Fig. 3). Such spikes in total nitrogen and total phosphorus following applications of diquat appear to be associated with diquat-mediated cell lysis of macrophytes (Woodward et al. 2024). Subsequent increases in phytoplankton biomass have been seen in ponds and shallow lakes: when weed control programs including herbicides have been used to manage aquatic macrophytes, their removal can lead to more turbid phytoplankton-dominated systems (an alternative stable ecosystem state; Scheffer 1998) which are less desirable than clear water systems. An increase in phytoplankton biomass could be an unintended consequence of diquat treatments as noted in some field applications (e.g., Parsons et al. 2007), but has not otherwise been well documented.

Chlorophyte phytoplankton dominated the mesocosms regardless of treatment. Differences in sensitivity to diquat among algal species have previously been reported (Peterson et al. 1997). Chlorophytes appear to be the least sensitive relative to cyanobacteria and diatoms (Peterson et al. 1997; Nagai 2019), including the same species found dominating in the present study at the highest concentrations. The diquat EC_50_ (96-h growth test based on changes in chlorophyll a) for a chlorophyte species in culture was estimated to be 64–95 µg/L (Fairchild et al. 1997), concentrations that were still present at day 7 in the higher application mesocosms. Diquat reduced the diversity of the phytoplankton assemblage at all concentrations (Fig. 4), suggesting tolerance of only a few taxa, and significantly changed the overall community structure even when species diversity and total algal biomass concentrations returned to control levels within a month of application. Such changes in community composition are commonly reported in studies on the effects of herbicides on algae in aquatic systems (e.g., (Smedbol et al. 2018), even at low doses (e.g., Pannard et al. 2009)) as seen in the present study. We suspect that direct effects of diquat and the resulting increase in dissolved bioavailable nutrients with macrophyte degradation were the two predominant factors affecting the change in phytoplankton community in our mesocosms.

### Amphipods

To our knowledge, the current study is the first to evaluate chronic effects of diquat on invertebrates, a data gap identified in a 2008 California risk assessment of diquat (Siemering et al. 2008). Results from our mesocosm study demonstrated that *H. azteca* is sensitive to chronic exposure to diquat resulting from a single application at concentrations below the label rate: LC_50s_ ranged from 170 µg/L at 2 weeks to 155 µg/L at 6 weeks (i.e., 15–17% of application rate), with complete mortality observed at concentrations of 1016 µg/L (i.e., 100% application rate). Acute laboratory studies show that *H. azteca* is particularly sensitive to diquat. For example, Wilson and Bond (1969) found that the median tolerance limits (i.e., LC_50s_) of diquat to *H. azteca* were 580 µg/L, 120 µg/L and 48 µg/L for 24-, 48- and 96-h assays, respectively, which are lower than expected based on our 2-week LC_50_ of 170 µg/L. However, the same study also found that diquat toxicity decreased in the presence of sediment and reported a 96-h median tolerance limit of 680 µg/L (i.e., four times higher than our 2-week LC_50_) where sediment was present, suggesting the type of sediment and its proximity to amphipods may influence diquat toxicity. In Wilson and Bond (1969), amphipods were 2–4 orders of magnitude more sensitive to diquat compared to mayflies, caddisflies, tendipedids, damselflies and dragonflies, and studies reviewed in Campbell et al. (2000) also demonstrated that amphipods are among the most sensitive taxa to diquat. However, other taxa such as *Daphnia pulex* are also sensitive to diquat at environmentally relevant concentrations (reviewed in Campbell et al. 2000).

Our results are significant because they highlight that chronic toxicity of diquat to amphipods at environmentally relevant concentrations may occur even in the presence of sediment and plants. Amphipods that did survive diquat exposure were not negatively affected in terms of growth and reproduction and may have benefited from an increase in food resources (phytoplankton) on a per amphipod basis in treatments where survival was lower. Although the reproduction data were difficult to interpret due to their variability combined with limited replication, the results from a chronic diquat exposure with another planktonic crustacean species, *Daphnia magna*, showed that reproductive endpoints were not affected by a chronic exposure to diquat up to 100 µg/L (Kashian and Dodson 2002), lending support to our observation. To our knowledge, this is the only other study to identify chronic sublethal effects of diquat on invertebrates. Hence, further studies with increased replication are needed to provide more robust data regarding the effects of diquat on amphipod (and other crustacean) reproduction.

### Amphibians

No detrimental changes were detected in northern leopard frog tadpoles exposed to diquat for up to 6 weeks in an outdoor mesocosm. More specifically, tadpoles exposed to diquat in mesocosms had higher survival, were further developed and were larger than control tadpoles. In the mesocosms, the tadpoles had access to the degrading macrophytes as a food resource. In addition, phytoplankton biomass was higher in the treated mesocosms, particularly at day 7, compared to the controls. Tadpoles were observed consuming degrading macrophytes in diquat-treated mesocosms, whereas tadpoles in the control tanks were not obviously grazing on the living macrophyte tissues. The degrading macrophytes and increased algal biomass after the decay of the macrophytes likely provided additional and different nutritional resources used for growth and development that were not available for the control tadpoles. Cooke (1977) also found that *Rana temporaria* and *Bufo bufo* tadpoles caged in natural ponds treated with 1 mg/L of diquat were heavier and had more fragments of filamentous algae in their intestines than the control tadpoles. Cooke (1977) proposed that the tadpoles were capitalizing on the increased algal resources and potentially increased algal nutritional content in the diquat-treated ponds. However, for tadpoles in natural ecosystems treated with diquat, the loss of habitat and hiding sites with the removal of macrophytes may increase the risk of predation, and the degradation of plant material may cause decreases in DO.

Previous studies have reported a range of acute and chronic effects in amphibians exposed to diquat. In a laboratory exposure, there were no significant differences in survival, growth or development of *R. pipiens* tadpoles between diquat-treated tadpoles and control tadpoles after 55 d of continuous exposure to 532 µg/L diquat (i.e., Reward ®; Robinson et al. 2021). This may be because the controlled laboratory conditions and ad libitum feeding schedule removed the likelihood for food resource differences between treatments. Dial and Dial (1987) also found that *R. pipiens* tadpoles exposed to diquat at 10 mg/L for 16 d in the laboratory did not differ in survival or abnormalities compared to control tadpoles. However, they did find that when diquat exposure started at the embryo life stage, mortality increased for post-hatch tadpoles such that exposure to diquat at 5 mg/L for 13 d and 10 mg/L for 10 d resulted in 24.5% and 39.6% mortality, respectively (Dial and Dial 1987). Similarly, *Xenopus laevis* embryos and early larval life stages are more sensitive than prometamorphic tadpoles to the lethal effects of diquat (i.e., Midstream®), where 96-h LC_50_’s are 0.83 and 0.2 mg/L for embryos and premetamorphic larvae, respectively, and 11.8 mg/L for prometamorphic tadpoles (Babalola and van Wyk 2018). A following 21-d chronic study with *X. laevis* found low mortality after exposure to this formulated diquat product up to 0.14 mg/L but observed putatively non-thyroidal related delays in development and reduced front and hind limb lengths at 0.11 and 0.14 mg/L compared to controls (Babalola and Wyk 2021). Another study investigated the effects of diquat (i.e., Reward®) on northwestern salamander (*Ambystoma gracile*) larvae and found the LC_50_ after 96-h of exposure was 71.5 mg/L, but this decreased to 1.56 mg/L when larvae were subchronically exposed for 21 d (Moreton and Marlatt 2019). A significant decrease in body weight after 21 d of continuous exposure to 1.48 mg/L diquat was also found for *A. gracile*, where larval body weight was 74% less than controls (Moreton and Marlatt 2019). From these studies, we can see that diquat can affect survival and apical responses of amphibians, but life stage, exposure duration and species differences need to be considered.

Finally, although diquat is a well-known substance that can induce oxidative damage (Saito 1986; Wolfgang et al. 1991), our 42-d mesocosm exposure found no significant differences in lipid peroxidation or protein carbonyl carboxylation measures between diquat treatments and controls. The requirement to pool liver samples within a mesocosm and the non-normality in the data reduced the resolution of our statistical analyses of oxidative stress markers, limiting the strength of our conclusions. However, in a separate laboratory study where continuous exposure to diquat at 532 µg/L was maintained for 8 weeks, *R. pipiens* experienced increased lipid peroxidation and protein carbonyl carboxylation, albeit with no obvious effects on fitness-relevant responses for the tadpoles (Robinson et al. 2021). Hence, negative effects on amphibians may occur with chronic and continuous or repeated exposure within an amphibian’s larval life stage (e.g., Moreton and Marlatt 2019; Robinson et al. 2021); however, these effects are not likely within current maximum label application rates and/or schedules, as shown with this mesocosm study (i.e., single application up to maximum application rate had no detrimental effects on tadpoles). Even so, the current label for Reward® suggests 2-week intervals between applications as needed, with no maximum number of applications within a growing season (Syngenta Canada Inc. 2015), and thus further research that fully simulates label recommended practices would be informative to assess the effects of chronic, recurring exposure to diquat on amphibians.

## Conclusions

Overall, our results suggest that diquat is lethal to both North American non-native and native plant species at concentrations well below the recommended application rate and that diquat may have negative effects on some non-target aquatic biota. Specifically, the generalist mode of action of diquat means that non-target native plant species exposed to diquat will also experience negative effects on growth and survival. Aquatic ecosystems are then more susceptible to predominately invasive macrophyte re-colonization, as invasive macrophytes are often able to outcompete native species (Tanner et al. 1990; Boylen et al. 1999), but not always (Parsons et al. 2007; Bugbee et al. 2020). Furthermore, there is evidence that with respect to invasive aquatic plants, lake-wide herbicide treatments have had larger effects on native plant species than the target of control, based on pre–post-treatment analyses (Mikulyuk et al. 2020) and that regular herbicide interventions in the field can lead to even stronger dominance of a non-native species (Thayer et al. 2024). Additionally, there is evidence that repeated applications of herbicides can increase tolerance in exposed aquatic plants (Dalton et al. 2013; Sesin et al. 2022). There is also a risk that a clear water macrophyte-dominated aquatic ecosystem that supports healthy fish populations by providing spawning sites, refuges and macrophyte-associated food resources (e.g., invertebrate and amphibian populations) could shift to a more turbid phytoplankton-dominated state, particularly under high nutrient conditions, that overall is less likely to support fish populations and more likely to lead to surface blooms of nuisance or potentially toxic algae (Pick 2016). The negative effects on the survival of amphipods at concentrations much less than the recommended diquat application rate may cause a shift in the invertebrate community and alteration of the food web. This shift has the potential to alter the quantity and quality of food for a variety of aquatic organisms, including fish species. However, extrapolation of our results to real-world scenarios is limited and should be conducted with caution, because a closed system and finite mesocosm exposure cannot fully replicate the scale and complexity (e.g., species diversity, indirect effects and long-term impacts, such as re-colonization of certain species and changes in ecosystem and habitat structure) of open, natural aquatic ecosystems; however, mesocosms do provide more realism than single species laboratory exposures (Fraser and Keddy 1997). Furthermore, the presence of unplanned species in our mesocosms (e.g., *Daphnia* spp., dragonfly larvae and fish hatched from eggs), may have influenced the results, albeit their occurrence was random with respect to mesocosm, and they are a relevant complexity as they are naturally occurring in aquatic ecosystems. In addition, our use of a formulated product corresponded to realistic environmental applications; however, formulations contain additives as well as the active ingredient. We used the formulation that is currently registered for use in Canada; but other formulations may have different additives and thus cause different effects. Nevertheless, the recommended application rate for diquat may be higher than necessary to control invasive aquatic plant species, particularly in non-turbid, shallow systems. The label rate is based on surface area (18.3 L/ha for waterbodies ≤ 1.5 m); however, the exposure concentration for biota is dependent on the volume of a given waterbody. Thus, label rate applications of diquat will result in diquat concentrations that vary depending on waterbody depth. In addition, diquat concentrations may be higher in localized treated areas with minimal mixing potential (e.g., protected inlet/bay within a lake, or smaller closed pond system) and lower in turbid, well-mixed areas (e.g., area with an inflow, current, or wave disturbance). A lower label rate and one that outlines maximum concentrations for specific systems and areas to be treated could also mitigate potential negative effects on invertebrates, providing effective control of invasive aquatic plants while reducing effects on non-target aquatic biota. However, further studies and field testing would be beneficial to compare to our mesocosm results, particularly in aquatic systems with varying amounts of suspended solids (e.g., clay particles or organic matter), to more fully understand the neutralization of diquat in differing aquatic systems.

## Supplementary Information

Below is the link to the electronic supplementary material.

Supplementary file1 (DOCX 1143 KB)

Supplementary file2 (XLSX 3418 KB)

## Data Availability

The datasets generated and analyzed during the current study are available as an Online Resource: Online Resource 2.
