# Supplementary material for: Effects of the Aquatic Herbicide Diquat on Non-Target Aquatic Biota: A Mesocosm Study
Source: Arch Environ Contam Toxicol. 2025 Sep 26;89(4):451–68. doi: 10.1007/s00244-025-01161-6 (PMC12665633; doi:10.1007/s00244-025-01161-6)
Supplement: Supplementary file 1 — Supplementary file1 (DOCX 1143 KB) [file 244_2025_1161_MOESM1_ESM.docx]

**Online Resource 1. Supplemental Information**

**Effects of the aquatic herbicide diquat on non-target aquatic biota – a mesocosm study**

**Archives of Environmental Contamination and Toxicology**

Dalton, R.L.^a,b,1*^, Robinson, S.A.^a,c*^, Bartlett, A.J.^d^, Sesin, V.^e^, Ben Othman, H.^b^, Carpenter, D.J. ^c^, Morrill, A. ^c^, Prosser, R.^f^, Rohonczy, J. ^a^, Pick, F.R.^b^

^a.^ Department of Biology, Carleton University, 1125 Colonel By Drive, Ottawa, ON, Canada K1S 5B6

^b.^ Department of Biology, University of Ottawa, 30 Marie Curie Private, Ottawa, ON, Canada K1N 6N5

^c.^ Wildlife and Landscape Science Directorate, Environment and Climate Change Canada, 1125 Colonel By Drive, Ottawa, ON, Canada K1A 0H3

^d.^ Water Science and Technology Directorate, Environment and Climate Change Canada, 867 Lakeshore Road, Burlington, ON, Canada L7S 1A1

^e.^ Institute of Environmental Sciences, University of Kaiserslautern-Landau, 76829 Landau, Germany

^f.^ School of Environmental Sciences, University of Guelph, 50 Stone Road East, Guelph, ON, Canada N1G 2W1

^1.^ Science Reporting and Assessment Directorate, Environment and Climate Change Canada, Gatineau, QC, Canada (Present address)

***Corresponding Author:** Stacey A. Robinson, email: [Stacey.Robinson@ec.gc.ca](mailto:Stacey.Robinson@ec.gc.ca)

**Table S1:** Linear models of mesocosm algal Shannon diversity index as dependent on experimental diquat treatment concentration, observation day (day zero, seven, or 41), and/or their interaction, compared using Akaike’s Information Criterion corrected for small sample sizes (AICc). The model with “1” listed as the sole predictor represents the single-intercept, i.e. the null, model.

| Model | Degrees of freedom | Log-likelihood | AICc | ΔAICc |
| --- | --- | --- | --- | --- |
| Shannon ~ Day + Treatment + Day:Treatment | 19 | -18.59 | 86.2 | 0 |
| Shannon ~ Day + Treatment | 9 | -44.41 | 109.1 | 22.9 |
| Shannon ~ Day | 4 | -55.9 | 120.27 | 34.07 |
| Shannon ~ 1 | 2 | -87.66 | 179.46 | 93.26 |
| Shannon ~ Treatment | 7 | -82.1 | 179.58 | 93.38 |

**Table S2:** Comparison of *Hyalella* *azteca* amphipod survival models based on expected log-predictive density (ELPD) estimated by direct leave-one-out cross-validation (LOO-CV). Model descriptions indicate the modeled hypothetical relationship between diquat concentration and amphipod survival probability on the logit scale. Higher ELPD values indicate models estimated to be more predictive of held-out data. All models included the random effect of tank.

| Survival model | ELPD | ELPD standard error | Difference in ELPD | ELPD difference standard error |
| --- | --- | --- | --- | --- |
| Quadratic polynomial relationship with diquat | -378.18 | 24.80 | 0 | 0 |
| Linear relationship with diquat | -453.5 | 17.04 | -75.31 | 16.77 |
| No effect of diquat | -497.2 | 14.53 | -119.02 | 18.16 |

**Table S3:** Model-estimated lethal concentrations (LC) of diquat (μg/L) causing 50% (LC_50_), 25% (LC_25_), and 10% (LC_10_) mortality of caged *Hyalella* *azteca* amphipods exposed for two, four, and six weeks. The limiting doses of stimulation (LDS) are also provided, i.e. the estimated diquat concentrations at which survival returns to the level observed at the control concentration, after the initial increase in survival probability. Estimates are medians of MCMC parameter samples, and 95% credible intervals (highest density continuous intervals) are provided in parentheses.

| Exposure duration | LC50 | LC25 | LC10 | LDS |
| --- | --- | --- | --- | --- |
| Two weeks | 170.10  (140.38 – 200.96) | 128.17  (99.33 – 158.49) | 103.21  (71.51 – 135.23) | 83.45  (46.85 – 120.29) |
| Four weeks | 162.26  (126.81 – 200.33) | 125.21  (92.16 – 165.57) | 105.6  (68.38 – 146.02) | 92.52  (51.58 – 134.47) |
| Six weeks | 155.42  (114.96 – 200.05) | 118.95  (79.21 – 163.4) | 101.28  (60.42 – 146.56) | 89.97  (47.30 – 136.07) |

**Table S4:** Summaries of parameters from the best-fitting binomial generalized linear mixed effect model (GLMM) of the proportion of juvenile amphipods (*Hyalella azteca*) at week six following experimental treatment with diquat. Models included an observation-level (cage) random effect as the models were otherwise overdispersed. The proportion of juvenile amphipods increased with increasing (log_2_-transformed) diquat concentration. SD: standard deviation.

| Parameter | Estimate | Standard error | Z value | *p* value |
| --- | --- | --- | --- | --- |
| Intercept | -0.314 | 0.323 | -0.97 | 0.332 |
| log_2_(Diquat concentration + 1) | 0.171 | 0.054 | 3.177 | 0.001 |
| Random effect (cage) SD | 0.917 |  |  |  |

**Table S5**: Summaries of parameters from best-fitting models of northern leopard frog (*Rana* [*Lithobates*] *pipiens*) tadpole survival, as well as life history- and condition-related responses. Survival was modeled using a generalized linear mixed effects model (GLMM) with a binomial family response (logit link); all other described models are linear mixed effects models. Tadpole development (Gosner stages/day), mass (in grams), and hepatosomatic index (HSI) were log_2_-transformed prior to modeling. The best-fitting model of tadpole sex ratios is not summarized because the null model was preferred over those models including any treatment or Gosner stage effects. Fixed effect parameter tests of significance are T-tests for LMMs and Wald Z-tests (no degrees of freedom considered) for GLMMs. SD: standard deviation.

| Response | Parameter | Estimate | Standard error | Test statistic (*Z* or *T*) | Degrees of freedom | *p* value |
| --- | --- | --- | --- | --- | --- | --- |
| Survival | Intercept | 1.24 | 0.30 | 4.07 | n/a | < 0.001 |
|  | log_2_(Diquat concentration) | 0.10 | 0.04 | 2.21 | n/a | 0.027 |
|  | Random effect (tank) SD | 0.34 |  |  |  |  |
| Development | Intercept | -3.45 | 0.23 | -15 | 30.42 | < 0.001 |
|  | log_2_(Diquat concentration) | 0.19 | 0.03 | 5.99 | 29.96 | < 0.001 |
|  | Random effect (tank) SD | 0.48 |  |  |  |  |
|  | Residual SD | 0.94 |  |  |  |  |
| Mass | Intercept | -15.10 | 0.68 | -22.21 | 376.25 | < 0.001 |
|  | Stage | 0.90 | 0.04 | 23.62 | 371.86 | < 0.001 |
|  | Stage^2^ | -0.01 | 0.001 | -25.24 | 370.68 | < 0.001 |
|  | log_2_(Diquat concentration) | 0.03 | 0.01 | 3.13 | 33.21 | < 0.001 |
|  | Random effect (tank) SD | 0.14 |  |  |  |  |
|  | Residual SD | 0.34 |  |  |  |  |
| HSI | Intercept | -7.15 | 0.12 | -61.83 | 168.30 | < 0.001 |
|  | Stage | -0.05 | 0.02 | -2.32 | 320.79 | 0.02 |
|  | Stage^2^ | 0.01 | 0.001 | 7.57 | 362.56 | < 0.001 |
|  | Random effect (tank) SD | 0.16 |  |  |  |  |
|  | Residual SD | 0.62 |  |  |  |  |


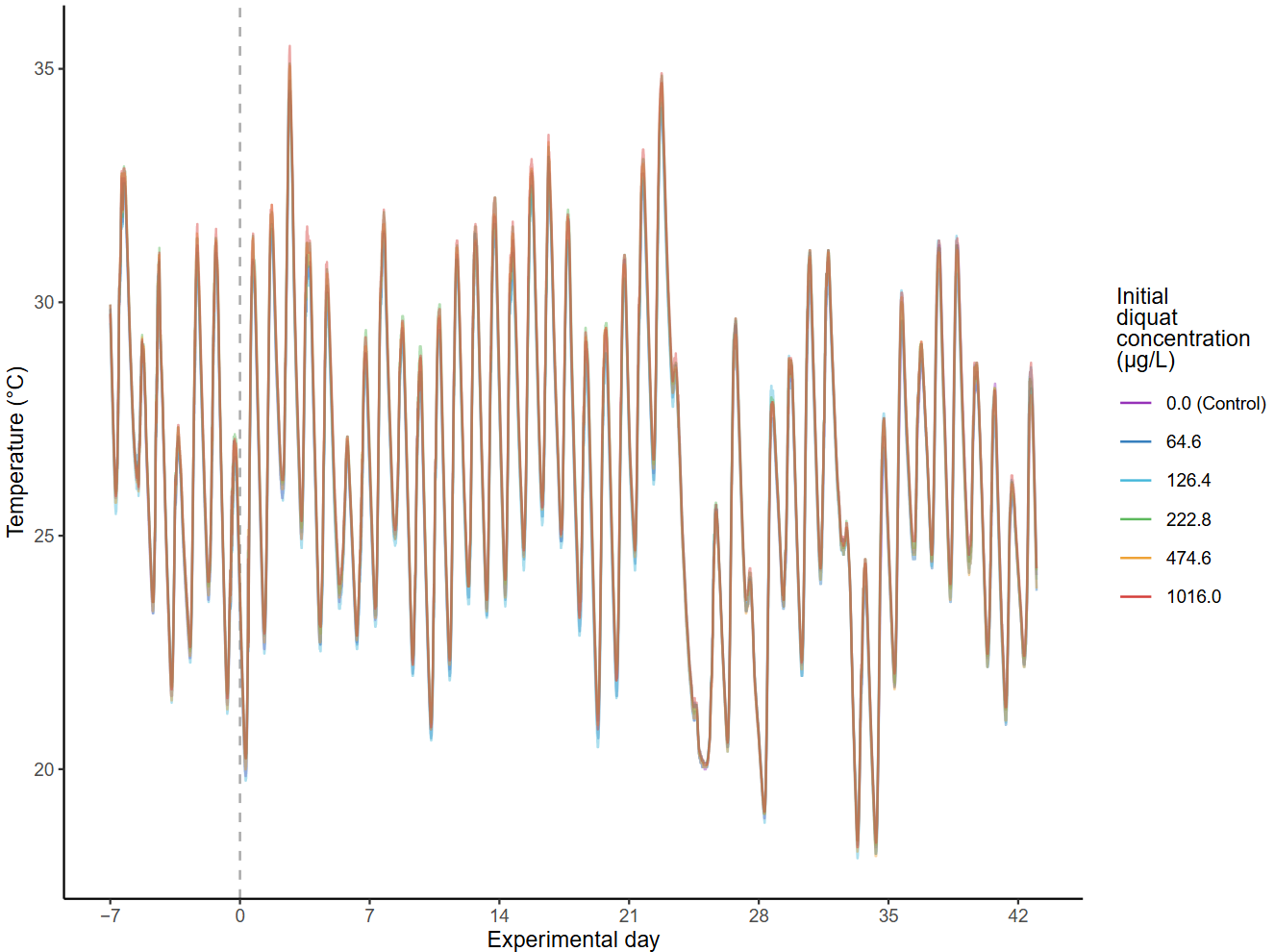


**Figure S1**. Average temperatures (˚C) in each treatment recorded by HOBO data loggers. Initial diquat concentrations are average measured concentrations one hour after dosing. Recordings were taken at five-minute intervals from 12:00 AM on July 13^th^, 2016 to 11:50 PM on August 31^st^, 2016. The dashed grey vertical line represents the date of the application of the experimental diquat treatments (July 20^th^).


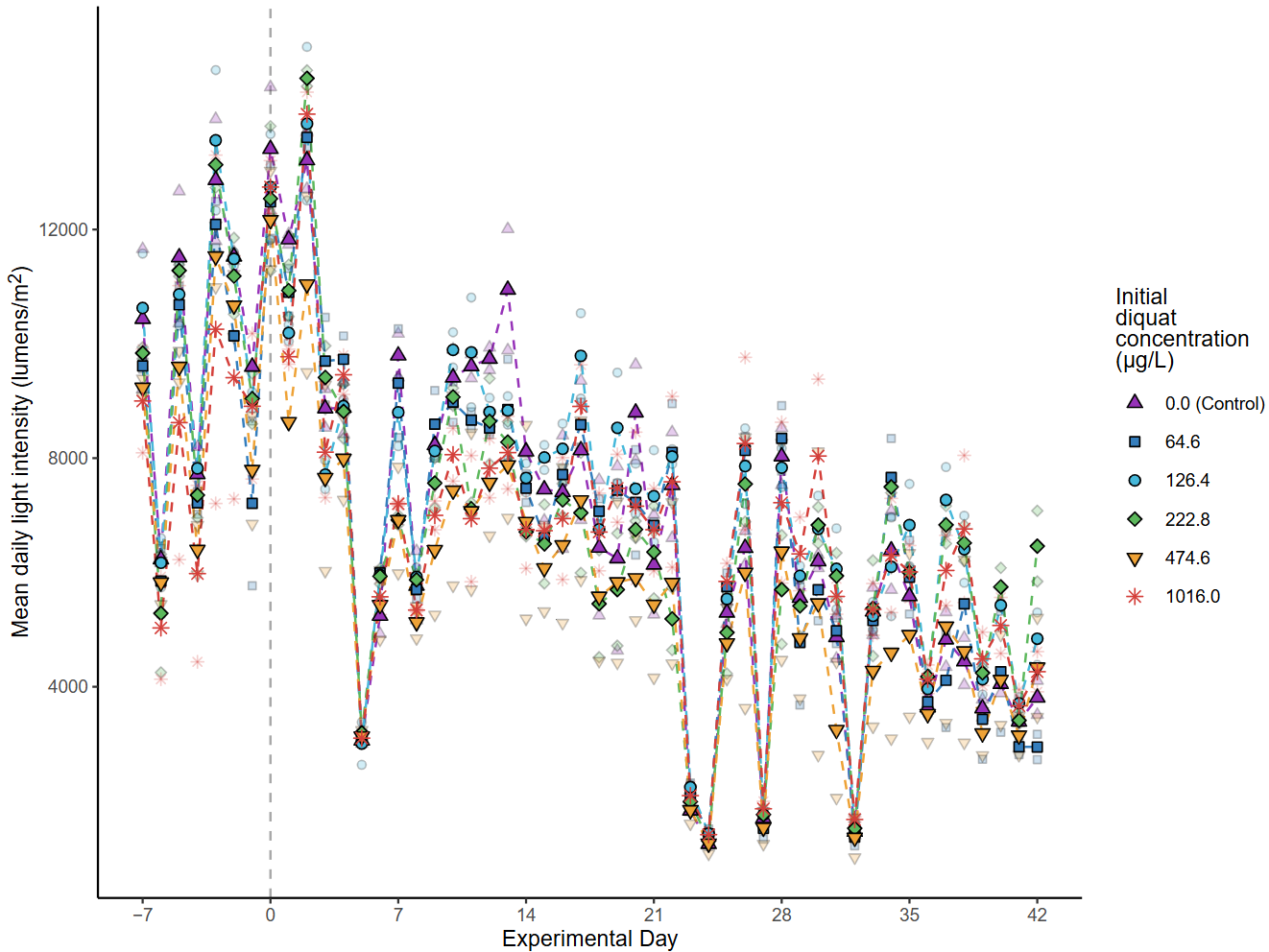


**Figure S2:** Average light intensity reaching the bottom of tanks in each of the experimental treatments from July 13^th^, 2016 to August 31^st^, 2016 recorded by HOBO data loggers. Translucent points represent individual mesocosm averages and are slightly randomly jittered horizontally to reduce point overlap, while solid points represent overall daily averages. The dashed grey vertical line represents the date of the application of the experimental diquat treatments (July 20^th^). Initial diquat concentrations are average measured concentrations one hour after dosing. Note that daily averages of light intensity are across all hours and not just daylight hours, and therefore the negative trend in light intensity as the summer progresses corresponds to the shortening span of daylight hours within each day. There was no significant difference among the treatments when tank was included with date as a random effect in models; otherwise, a single tank in the 474.6 μg/g initial concentration group with consistently low values brings that group’s average down.


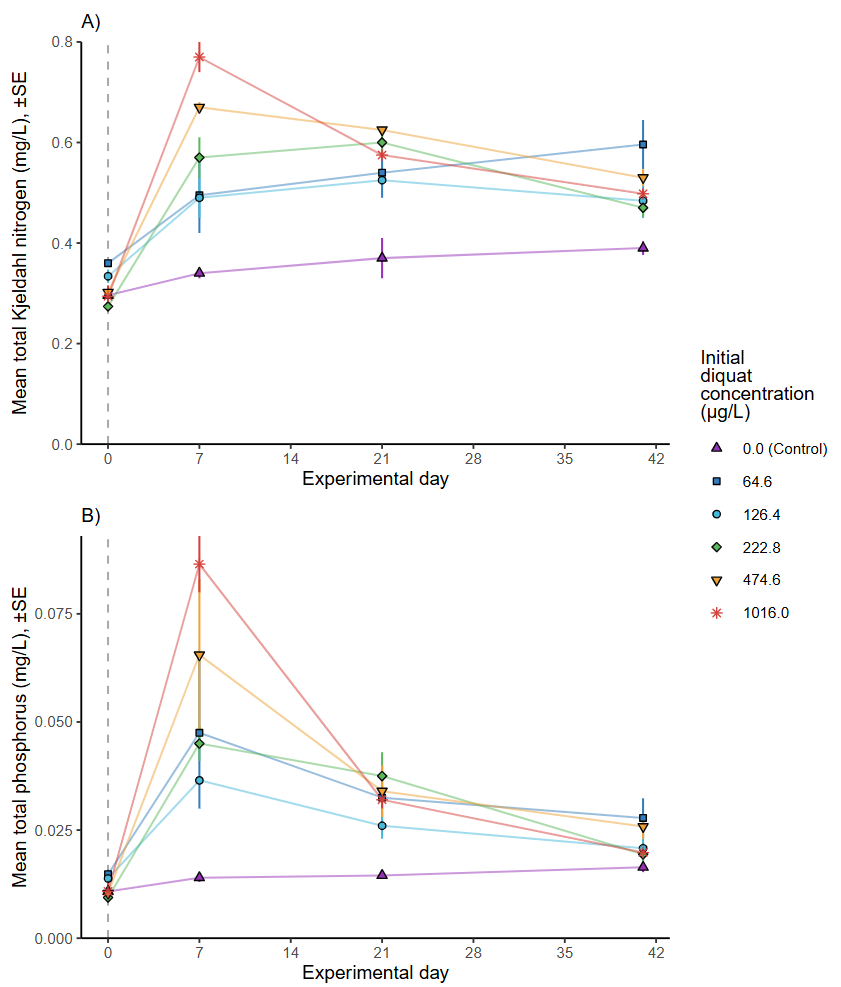


**Figure S3**. Average total Kjeldahl nitrogen (A) and total phosphorus (B) measured in mesocosms on four different dates across experimental treatments. Initial diquat concentrations are average measured concentrations one hour after dosing. The dashed grey line indicates the date of exposure to diquat (July 20^th^).


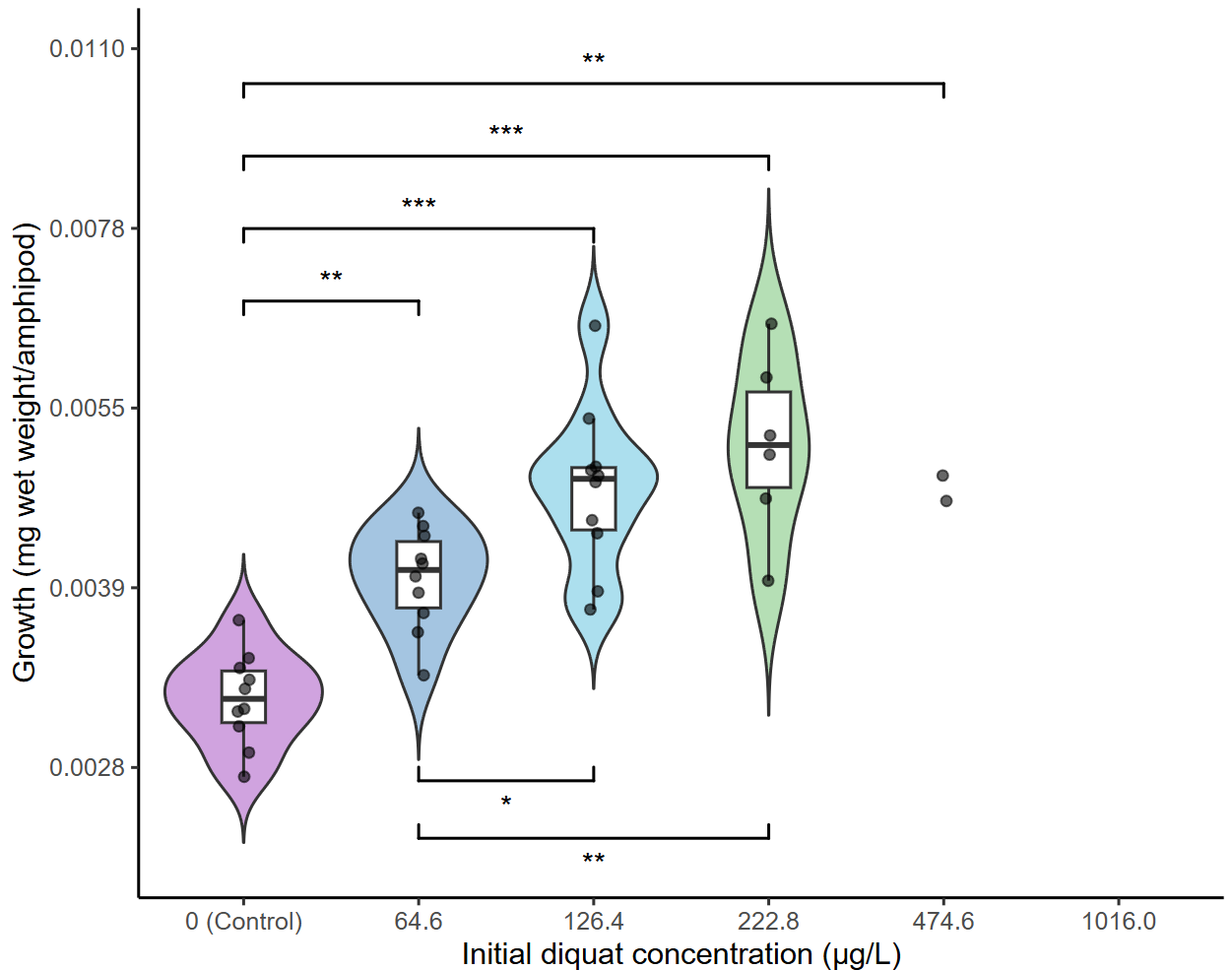


**Figure S4:** Growth of amphipods (*Hyalella azteca*) at six weeks following experimental treatment with diquat, measured as mg wet weight/amphipod in a cage. Brackets indicate groups whose means were identified as significantly different according to a Tukey’s HSD post-hoc test, following an ANOVA (*p* < 0.05 = *; *p* < 0.01 = **; *p* < 0.001 = ***). While growth on the y-axis is untransformed, note that the axis itself is on the log-scale. No growth is shown for the 1016.0 μg/L treatment because there were no surviving amphipods at six weeks for that concentration of diquat. Initial diquat concentrations are average measured concentrations one hour after dosing.


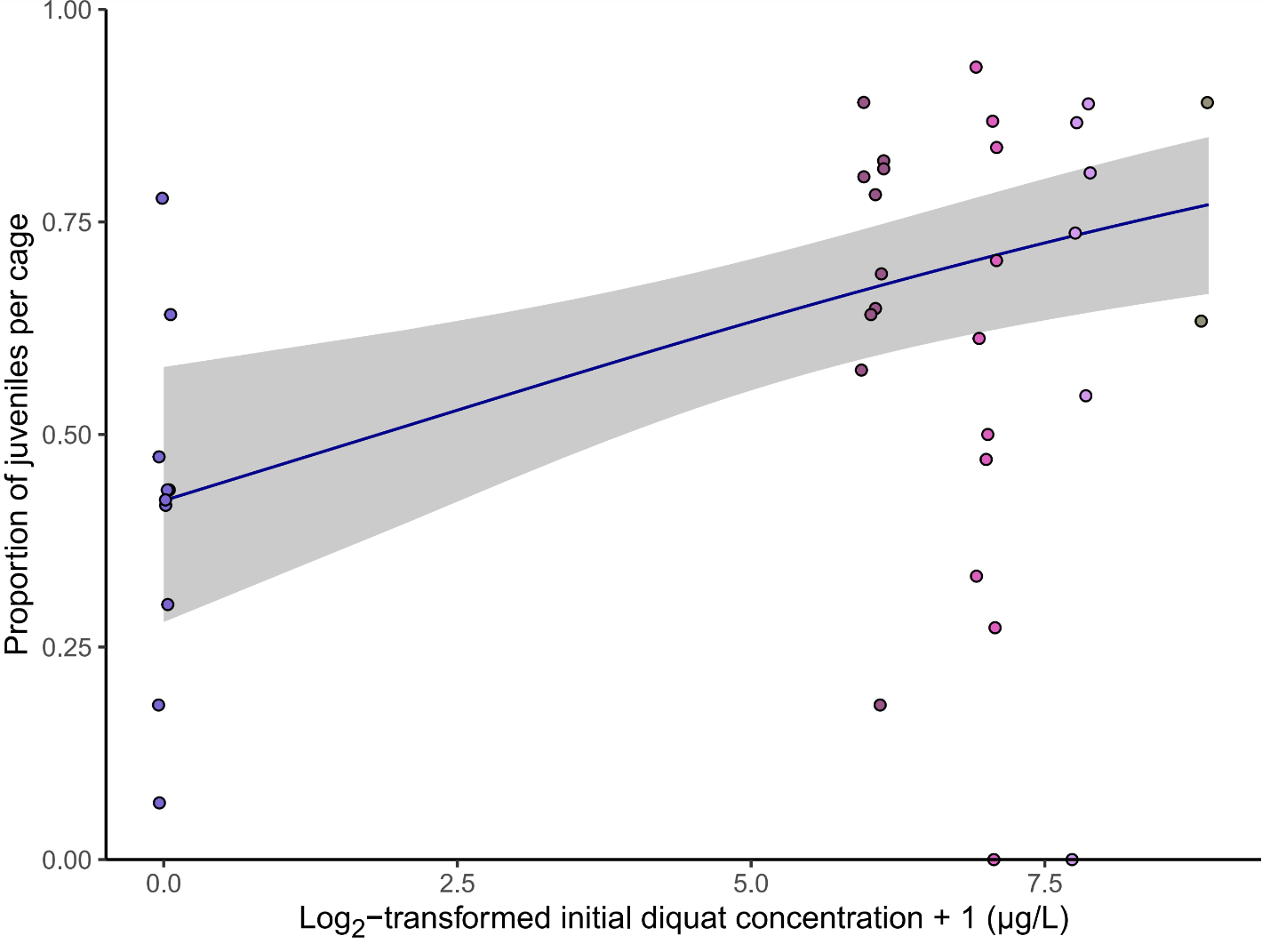


**Figure S5.** Proportions of juvenile amphipods (*Hyalella azteca*) in each cage at six weeks following experimental treatment with diquat. Best-fitting binomial model predictions are indicated with the smooth line, and the shaded region represents the 95% confidence interval. Model predictions are conditional, i.e., they represent predictions for a single (average) cage. Points are randomly horizontally jittered to help reduce overlap. No proportions are shown for the 1016.0 μg/L treatment because there were no surviving amphipods at six weeks for that initial concentration of diquat.


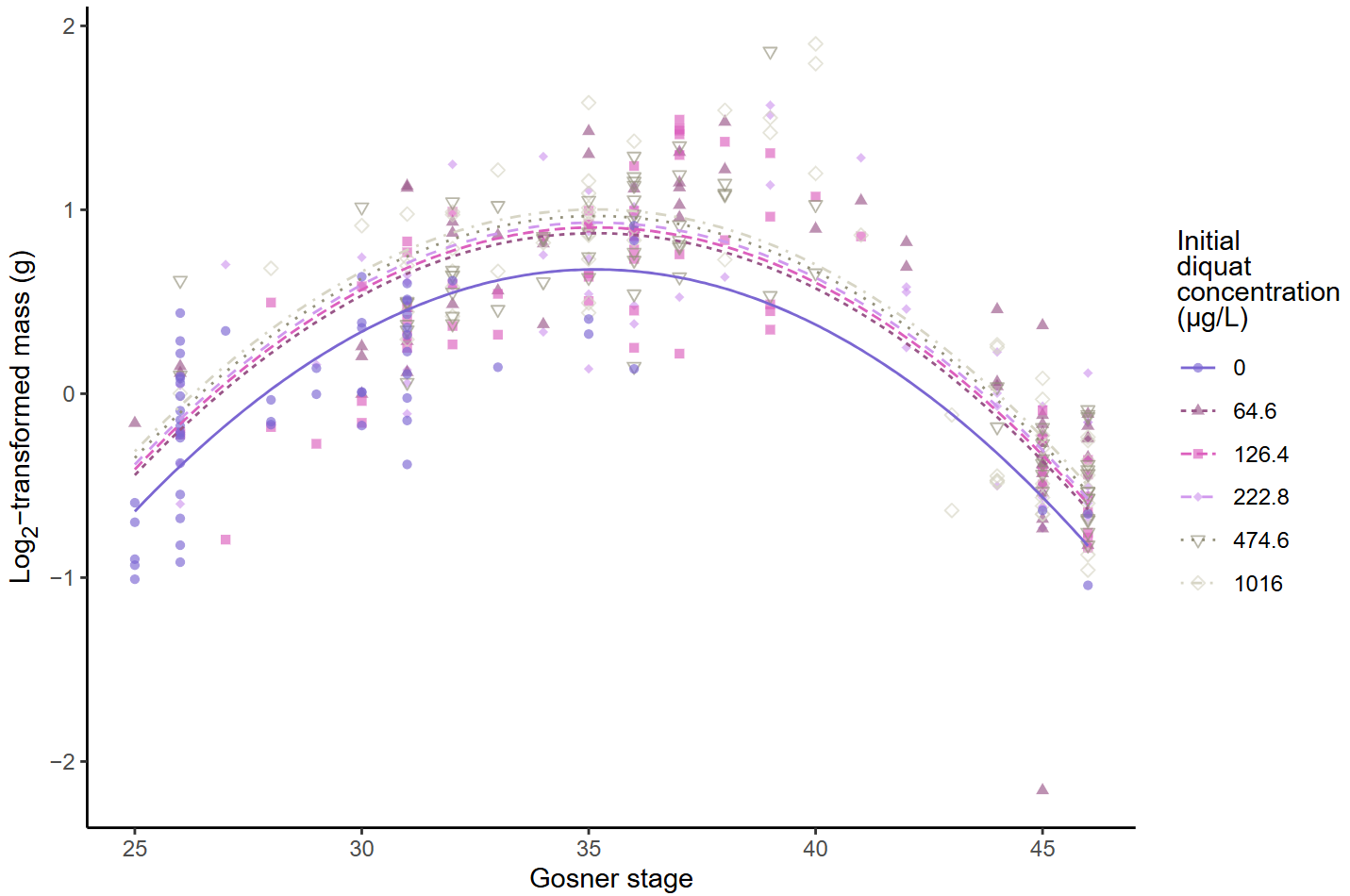


**Figure S6**: The mass (log_2_-transformed) of individual tadpoles (*Lithobates pipiens*) follows a parabolic relationship with increasing Gosner stage, where mass initially increases with development, but later decreases. After controlling for this relationship, transformed mass additionally increases with (log_2_-transformed) diquat concentration. Points represent the observed masses of individual tadpoles. Fitted lines are average predictions of tadpole mass for each diquat concentration from the best-fitting linear mixed-effects model.

***Formulation and evaluation of Bayesian amphipod (Hyalella* *azteca) survival models***

All amphipod survival models were programmed in the Stan language (Stan Development Team, 2024) and were run through the cmdstanr R interface (version 0.5.3; Gabry and Češnovar, 2022).

Numbers of surviving amphipods in each tank (*S_i_*) were modeled as following a binomial distribution:

$$S_{i} \sim\text{Binomial}({N_{i},p}_{i})$$

where *p_i_* represents the probability of individual amphipod survival in tank *i* and *N_i_* represents the total number of initial amphipods in each tank. Within-tank survival probability in the full model was then equated as:

$$logit\left( p_{i} \right)=\alpha_{j}+\beta_{1j}{C_{i}}^{2}+\beta_{2j}C_{i}+\lambda_{i}$$

where *α_j_* represents the week-specific intercept on the logit scale for week *j*, *C_i_* is the log_2_-transformed concentration of diquat to which amphipods in tank *i* were exposed, *λ_i_* is the tank-level random effect, and the two week-specific *β* terms describe the varying effect of transformed diquat on survival as the concentration itself changes. In other words, conditional on a particular tank, the effect of transformed diquat follows a quadratic function on the logit scale; this translated to a bell-shaped relationship on the probability scale, describing survival as initially increasing, but then later decreasing, with increasing diquat concentration. This full model was compared using exact leave-one-out cross validation to a simpler model with only a linear effect of transformed diquat concentration (on the logit scale; i.e. survival only increasing or only decreasing with increasing diquat), as well as to a more “null” model which only included the tank-level random effect and the week-specific intercept (i.e. no effect of diquat). Model comparisons were based on expected log pointwise predictive densities (ELPDs) calculated on the held-out data (Vehtari et al., 2017). Models were considered to provide meaningfully different predictive performances on held-out data when the estimated difference in their ELPDs was greater than four, and when 2× the standard error on the ELPD difference was less than the ELPD difference itself (McLatchie et al., 2023, Sivula et al., 2023).

The *α* and *β* parameters used weakly informative (on the logit-scale) normally distributed priors with standard deviations of 1.5 and 5, respectively. The *β_1_* parameter was additionally constrained to be negative, to ensure that any estimated relationship between survival and diquat would be concave down (i.e. a half-normal distribution prior). Concentrations of diquat being incremented by one prior to log_2_-transformation meant that conditional intercepts (*α_j_*) represented the estimated survival for the control treatment for each week on the logit scale. The standard deviation of the normal distribution describing the random effect *λ* was given a weakly-informative exponential prior with rate parameter equal to one (McElreath, 2020).

The model with both *β_1_* and *β_2_* parameters (i.e. the model with a quadratic relationship between logit-transformed survival and log_2_-transformed diquat concentration) allowed the estimation of different lethal concentrations of diquat (LC_50_, LC_25_, LC_10_) as well as the limiting dose of stimulation (i.e. the estimated diquat concentrations at which survival returns to the level observed at the control concentration) using solutions to the quadratic equation. These were calculated in the generated quantities block of the Stan model so that their estimates would be provided as whole posterior distributions, and to ensure point estimates could be summarized with uncertainties.

Models were fit using 1,500 warm-up MCMC iterations and 1,000 sampling iterations in each of three chains (total MCMC samples = 3,000). Convergence diagnostics were checked to ensure that all parameters had *R̂* values below 1.02 (indicating good mixing of MCMC chains), and that parameter effective sample sizes (ESS) exceeded a total of 100 per MCMC chain (Vehtari et al., 2021).

**References**

Gabry J, Češnovar R (2022) cmdstanr: R Interface to 'CmdStan'. <https://mc-stan.org/cmdstanr/>, <https://discourse.mc-stan.org>.

McElreath, R (2020) Statistical rethinking: a Bayesian course with examples in R and Stan, 2^nd^ ed. CRC Press, Boca Raton, Florida.

McLatchie, Y, Rögnvaldsson, S, Weber, F, and Vehtari, A (2023) Advances in projection predictive inference. arXiv:2306.1558. URL: <https://doi.org/10.48550/arXiv.2306.15581>.

Sivula, T, Magnusson, M, Matamoros, AA, and Vehtari, A (2023) Uncertainty in Bayesian leave-one-out cross-validation based model comparison. arXiv:2008.10296. URL: <https://doi.org/10.48550/arXiv.2008.10296>.

Stan Development Team (2024) Stan Modeling Users Guide and Reference Manual. <http://mc-stan.org>

Vehtari, A, Gelman, A, Gabry, J (2017) Practical Bayesian model evaluation using leave-one-out cross-validation and WAIC. Stat. Comput. 27, 1413–1432. <https://doi.org/10.1007/s11222-016-9696-4>

Vehtari, A, Gelman, A, Simpson, D, Carpenter, B, Bürkner, P-C (2021) Rank-normalization,

folding, and localization: an improved R̂ for assessing convergence of MCMC (with Discussion). Bayesian Anal16, 667–718. <https://doi.org/10.1214/20-BA1221>
